# Supplementary material for: Identification of photoperiod-induced specific miRNAs in the adrenal glands of Sunite sheep (Ovis aries)
Source: Front Vet Sci. 2022 Jul 22;9:888207. doi: 10.3389/fvets.2022.888207 (PMC9354845; doi:10.3389/fvets.2022.888207)
Supplement: Supplementary file 1 [file Data_Sheet_1.ZIP › Supplementary file/miRDeep2 file.zip!/miRDeep2 file/Novel_Structure/SPLP42A2/Novel_474.pdf]

Provisional ID	:	NC_019459.2_4880
Score total	:	4.3
Score for star read(s)	:	3.9
Score for read counts	:	-2.8
Score for mfe	:	1.7
Score for randfold	:	1.6
Score for cons. seed	:	
Total read count	:	6
Mature read count	:	5
Loop read count	:	0
Star read count	:	1

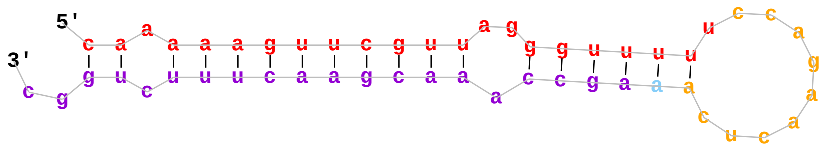

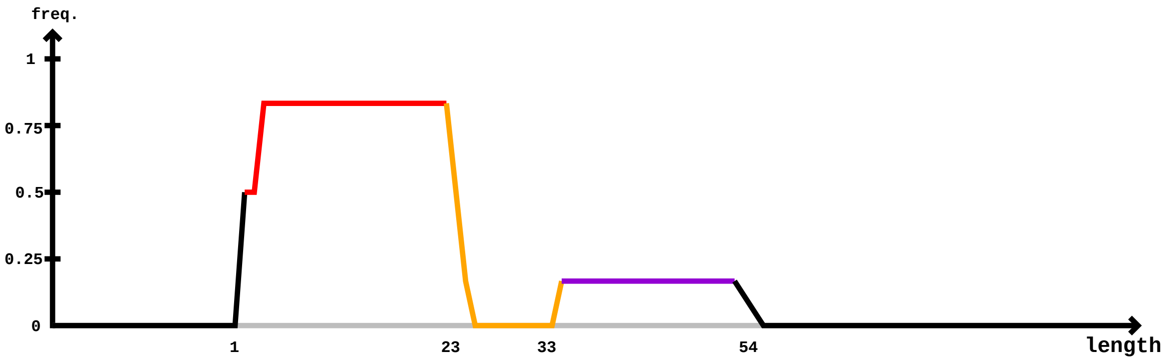


```
                                    Mature                              Star
5'- ugcaaguauuggguuggccaaaaaguucguuaggguuuuuccagaacucaaagccaaacgaacuuucuggcuuaaccaauacuucauaaguguuagucgcucggucgugug   -3'   obs
    ugcaaguauuggguuggccaaaaaguucguuaggguuuuuccagaacucaaagccaaacgaacuuucuggcuuaaccaauacuucauaaguguuagucgcucggucgugug         exp
    ((.(((((((((...(((((.((((((((((..((((((...........)))))).))))))))))).)))))...)))))))))))..((((.....))))......... reads  mm  sample
    ..................caaaaaguucguuCggguuuuu.......................................................................   2     1   seq
    ..................caaaaaguucguuUggguuuuuc......................................................................   1     1   seq
    ....................aaaaguucguuaggguuuuuc......................................................................   1     0   seq
    ....................aaaaguucAuuaggguuuuucc.....................................................................   1     1   seq
    ...................................................aCccaaacgaacuuucuggc........................................   1     1   seq
```
